# New Diagnostic Approach to Arrhythmogenic Cardiomyopathy: The Padua Criteria

**DOI:** 10.31083/j.rcm2310335

**Published:** 2022-10-10

**Authors:** Francesca Graziano, Alessandro Zorzi, Alberto Cipriani, Manuel De Lazzari, Barbara Bauce, Ilaria Rigato, Giulia Brunetti, Kalliopi Pilichou, Cristina Basso, Martina Perazzolo Marra, Domenico Corrado

**Affiliations:** ^1^Department of Cardiac, Thoracic and Vascular Sciences, University of Padua, 35128 Padova, Italy

**Keywords:** arrhythmogenic cardiomyopathy, cardiomyopathy, ventricular arrhythmias, cardiac magnetic resonance, diagnosis

## Abstract

Arrhythmogenic cardiomyopathy (ACM) is a rare heart muscle disease characterized by a progressive fibro-fatty myocardial replacement, ventricular arrhythmias, and increased risk of sudden cardiac death. The first diagnostic criteria were proposed by an International Task Force of experts in 1994 and revised in 2010. At that time, ACM was mainly considered a right ventricle disease, with left ventricle involvement only in the late stages. Since 2010, several pathological and clinical studies using cardiac magnetic resonance (CMR) imaging have allowed to understand the phenotypic expression of the disease and to reach the current idea that ACM may affect both ventricles. Indeed, left ventricular involvement may parallel or exceed right ventricular involvement. The main limitations of the 2010 criteria included the poor sensitivity for left ventricular involvement and the lack of inclusion of tissue characterization by CMR. The 2020 International criteria (the Padua criteria) were developed to overcome these shortcomings. The most important innovations are the introduction of a set of criteria for identifying left ventricular variants and the use of CMR for tissue characterization. Moreover, criteria for right ventricular involvement were also updated taking into account new evidence. According to the number of criteria for right and/or left ventricular involvement, the 2020 Padua criteria allows diagnosing three ACM phenotypic variants: right-dominant, biventricular and left-dominant. This review discusses the evolving approach to diagnosis of ACM, from the 1994 International Criteria to the 2020 Padua criteria.

## 1. Background

Arrhythmogenic cardiomyopathy (ACM) is a rare heart muscle disease pathologically characterized by a progressive replacement of the ventricular myocardium with fibro-fatty tissue, and clinically by life-threatening ventricular arrhythmias and sudden cardiac death (SCD) [1, 2].

At first, the disease was considered as only affecting the right ventricle (RV). The first report of ACM as heredo- familial disease was published in 1736 by Giovanni Maria Lancisi [3]. He described a family with disease recurrence in four generations, which occurred with palpitations, dilatation of the RV, heart failure and SCD. In 1982, Marcus *et al*. [4] introduced the term “arrhythmogenic right ventricular dysplasia”, by reporting 24 cases of adult patients with left bundle branch block morphology ventricular arrhythmias, in keeping with origin from an affected RV. It was considered a development defect of RV myocardium. Some years later, Thiene and colleagues [5] for the first time recognized this disease as a main cause of SCD in young people and athletes. Moreover, the post-mortem examination of the hearts brought the idea of a heart muscle disease developing after birth, because of the histopathological evidence of inflammation, degeneration, and necrosis foci, with progressive loss of myocardium. Therefore, the authors defined the disease “arrhythmogenic right ventricular cardiomyopathy” (ARVC) rather than “dysplasia”. The discovery of defects in genes encoding desmosomal proteins resulted in the definitively introduction of the ARVC in the WHO nomenclature and classification of cardiomyopathy [6]. Moreover, the absence of a single gold standard for reaching the diagnosis of ARVC led to the necessity of formal criteria aimed at facilitating and standardizing the clinical diagnosis.

## 2. The 1994 and 2010 International Task Force Criteria

In 1994 a group of experienced clinicians in cardiomyopathies published the first Task Force (TF) criteria [7]. The diagnosis was based on multiple parameters from six different categories, including global or regional dysfunction and structural alterations of the RV demonstrated on imaging, tissue characterization by endomyocardial biopsy (EMB), ECG repolarization abnormalities, ECG depolarization abnormalities, arrhythmias and family history. Each criterion was classified as “major” or “minor” according to its specificity for differentiating ARVC from conditions with an overlapping phenotype such as idiopathic right ventricular outflow tract (RVOT) ventricular tachycardia (VT) or biventricular dilated cardiomyopathy (DCM). It was proposed that the diagnosis of ARVC would be fulfilled by the presence from different groups of either two major criteria, or one major plus two minor criteria, or four minor criteria. At that time, ACM was mainly considered as a RV disease, with left ventricle (LV) involvement only in the late stages. Indeed, the morpho-functional RV abnormalities diagnostic criteria were met in presence of no or only mild LV impairment.

The 1994 TF criteria had some limitations. First, they resulted to be highly specific, but they lacked sensitivity when evaluating asymptomatic patients and relatives with early/minor ARVC. This is because the clinical experience was primary based on SCD victims and symptomatic index cases, with clear and severe manifestations of ACM [8]. Moreover, the criteria revealed faults in clinical application because of the qualitative and subjective assessment of the clinical parameters, rather than quantitative.

In 2010, the Revised International Task Force (ITF) criteria were published [9]. The organization in 6 different categories and the classification in major and minor criteria were maintained. In order to improve diagnostic accuracy, the 2010 ITF criteria provided quantitative imaging reference values for defining normal RV and various degree of structural and functional abnormalities, and also a quantitative histomorphometric definition of fibrofatty replacement of myocardium on EMB. In addition, ECG and ventricular arrhythmias criteria were updated, and the “family history” category was enriched with molecular genetic information [9, 10, 11, 12]. Another important element of novelty of the 2010 criteria is the introduction of “borderline” (1 major plus one minor or three minor criteria) and “possible” (1 major or two minor criteria) diagnostic categories.

## 3. A Critical Appraisal of the International Task Force Criteria

Since 2010, several pathological and clinical studies have allowed to better understand the phenotypic expression of the disease and to reach the current idea of a cardiomyopathy that can be biventricular or exceed either in RV involvement (ARVC) or in LV involvement (left-dominant or arrhythmogenic left ventricular cardiomyopathy (ALVC)) [13, 14]. Subsequently, the original term “ARVC” was replaced with the broader definition of “arrhythmogenic cardiomyopathy” (ACM) [15].

In 2019, a group of International Experts in cardiomyopathies published an extensive critical review [16] of the clinical performance of the 2010 criteria, emphasizing three major points:

(1) They lacked specific criteria for diagnosing left-sided variants;

(2) Cardiac magnetic resonance (CMR) has become crucial not only for assessing volumes, systolic function and wall motion abnormalities, but especially for tissue characterization using late gadolinium enhancement (LGE) technique, which is essential for diagnosing LV involvement that can be characterized by subepicardial fibrosis or fibro-fatty scars with or without ventricular wall motion abnormalities [17, 18];

(3) Genetic testing was considered a major diagnostic criterion also in probands, potentially allowing to reach the diagnosis also in absence of morpho-functional ventricular abnormalities or tissue changes. Instead, in all the other cardiovascular diseases, genetic testing is recommended in probands that already fulfill clinical diagnostic criteria.

In addition to these three points, the experts underlined that some RV criteria needed revision.

On the basis of this critical appraisal, in 2020 an international expert consensus document upgraded diagnostic criteria for ACM [19].

## 4. The Padua criteria

The 2020 International criteria (“Padua Criteria”) maintained the same organization in 6 sets of criteria, the differentiation of the criteria in major and minor depending on their specificity for diagnosing ACM and the three diagnostic categories (“definite”, “borderline” or “possible”). However, there were several innovations: (i) because ACM is a structural disease, at least one morpho-functional or structural criterion needed to be satisfied; (ii) tissue characterization through CMR was introduced; (iii) two different sets of criteria for identification of RV (updated criteria) and LV involvement (new criteria) were provided (Table 1, Ref. [19]).

**Table 1.** **The Padua criteria**.

	Criteria for RV involvement	Criteria for LV involvement
I. Morpho-functional ventricular abnormalities	***By 2D echocardiogram, CMR or angiography:***	***By 2D echocardiogram, CMR or angiography:***
	***Major***	***Minor***
	Regional RV akinesia, dyskinesia, or bulging *plus* one of the following:	Global LV systolic dysfunction (depression of LV EF or reduction of echocardiographic global longitudinal strain), with or without LV dilatation (increase of LV EDV according to the imaging test specific nomograms for age, sex, and BSA)
	-global RV dilatation (increase of RV EDV according to the imaging test specific nomograms for age, sex and BSA)	
	*or*	
	-global RV systolic dysfunction (reduction of RV EF according to the imaging test specific nomograms for age and sex)	
	***Minor***	***Minor***
	Regional RV akinesia, dyskinesia or aneurysm of RV free wall	Regional LV hypokinesia or akinesia of LV free wall, septum, or both
II. Structural myocardial abnormalities	***By CE-CMR:***	***By CE-CMR:***
	***Major***	***Major***
	Transmural LGE (stria pattern) of ≥1 RV region(s) (inlet, outlet, and apex in 2 orthogonal views)	LV LGE (stria pattern) of ≥1 Bull’s Eye segment(s) (in 2 orthogonal views) of the free wall (subepicardial or midmyocardial), septum, or both (excluding septal junctional LGE)
	***By EMB (limited indications):***	
	***Major***	
	Fibrous replacement of the myocardium in ≥1 sample, with or without fatty tissue	
III. ECG repolarization abnormalities	***Major***	***Minor***
	Inverted T waves in right precordial leads ($V_{1}$, $V_{2}$, and $V_{3}$) or beyond in individuals with complete pubertal development (in the absence of complete RBBB)	Inverted T waves in left precordial leads ($V_{4}$–$V_{6}$) without complete LBBB
	***Minor***	
	-Inverted T waves in leads V1 and V2 in individuals with completed pubertal development (in the absence of complete RBBB)	
	-Inverted T waves in V1, V2, V3 and V4 in individuals with completed pubertal development in the presence of complete RBBB	
IV. ECG depolarization abnormalities	***Minor***	***Minor***
	-Epsilon wave (reproducible low amplitude signals between end of QRS complex to onset of the T wave) in the right precordial leads (V1 to V3)	Low QRS voltages (<0.5 mV peak to peak) in limb leads (in the absence of obesity, emphysema, or pericardial effusion)
	-Terminal activation duration of QRS ≥55 ms measured from the nadir of the S wave to the end of the QRS, including R’, in V1, V2, or V3 (in the absence of complete RBBB)	
V. Ventricular arrhythmias	***Major***	***Minor***
	-Frequent ventricular extrasystoles (>500 per 24 hours), non-sustained or sustained ventricular tachycardia of LBBB non-inferior axis morphology	Frequent ventricular extrasystoles (>500 per 24 hours), non-sustained or sustained ventricular tachycardia with a RBBB morphology (excluding the “fascicular pattern”)
	***Minor***	
	-Frequent ventricular extrasystoles (>500 per 24 hours), non-sustained or sustained ventricular tachycardia of LBBB morphology with inferior axis (“RVOT pattern”)	
VI. Family history/genetics	***Major***	
	-ACM confirmed in a first-degree relative who meets diagnostic criteria	
	-ACM confirmed pathologically at autopsy or surgery in a first-degree relative	
	-Identification of a pathogenic or likely pathogenetic ACM mutation in the patient under evaluation	
	***Minor***	
	-History of ACM in a first-degree relative in whom it is not possible or practical to determine whether the family member meets diagnostic criteria	
	-Premature sudden death (<35 years of age) due to suspected ACM in a first-degree relative	
	-ACM confirmed pathologically or by diagnostic criteria in second-degree relative	

ACM, arrhythmogenic cardiomyopathy; BSA, body surface area; CE-CMR, cardiac enhanced-cardiac magnetic resonance; CMR, cardiac magnetic resonance; EDV, end diastolic volume; EF, ejection fraction; EMB, endomyocardial biopsy; LBBB, left bundle branch block; LGE, late gadolinium enhancement; LV, left ventricle; RBBB, right bundle branch block; RV, right ventricle; RVOT, right ventricular outflow tract. Adapted from Corrado *et al*. [19].

**Fig. 1.**
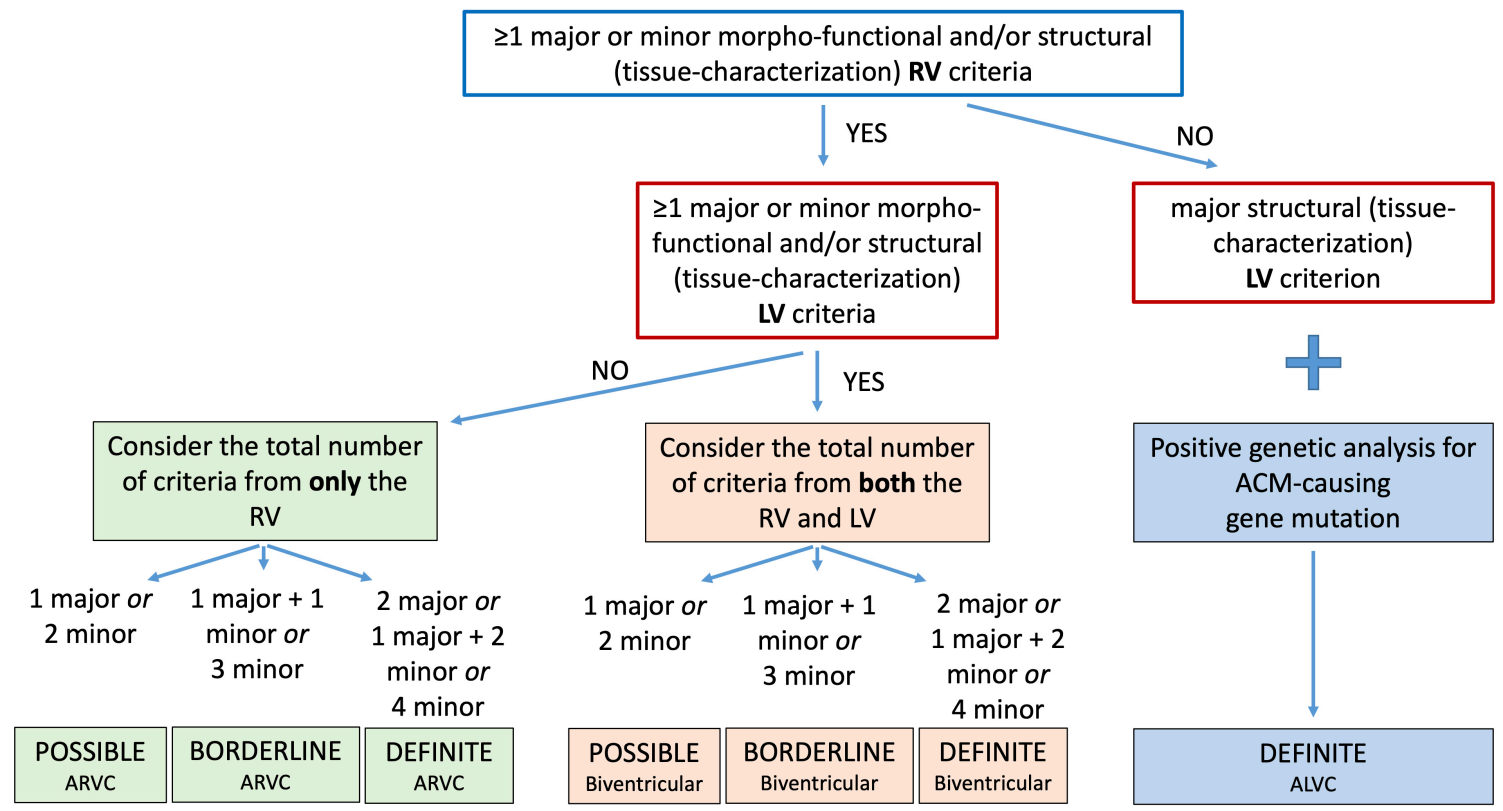
**Flowchart for phenotypic characterization of ACM**. The diagnosis of ACM requires at least 1 morpho-functional or structural abnormalities criterion, either major or minor. The diagnosis of the specific phenotypic variant depends on the ventricle interested on alterations (see text for details). Moreover, the likelihood of disease is defined by the combination of the major and minor criteria fulfilled. ACM, arrhythmogenic cardiomyopathy; ALVC, arrhythmogenic left ventricular cardiomyopathy; ARVC, arrhythmogenic right ventricular cardiomyopathy; LV, left ventricle; RV, right ventricle. Adapted from Corrado *et al*. [20].

Finally, according to the number of LV and RV criteria that are fulfilled, the 2020 criteria provide a classification of ACM in three different phenotypic variants: “dominant right” variant, which is the classical form with RV involvement; “biventricular disease” variant, with involvement of both ventricles; “dominant-left” variant, with involvement of only the LV (Fig. 1, Ref. [20]).

**Fig. 2.**
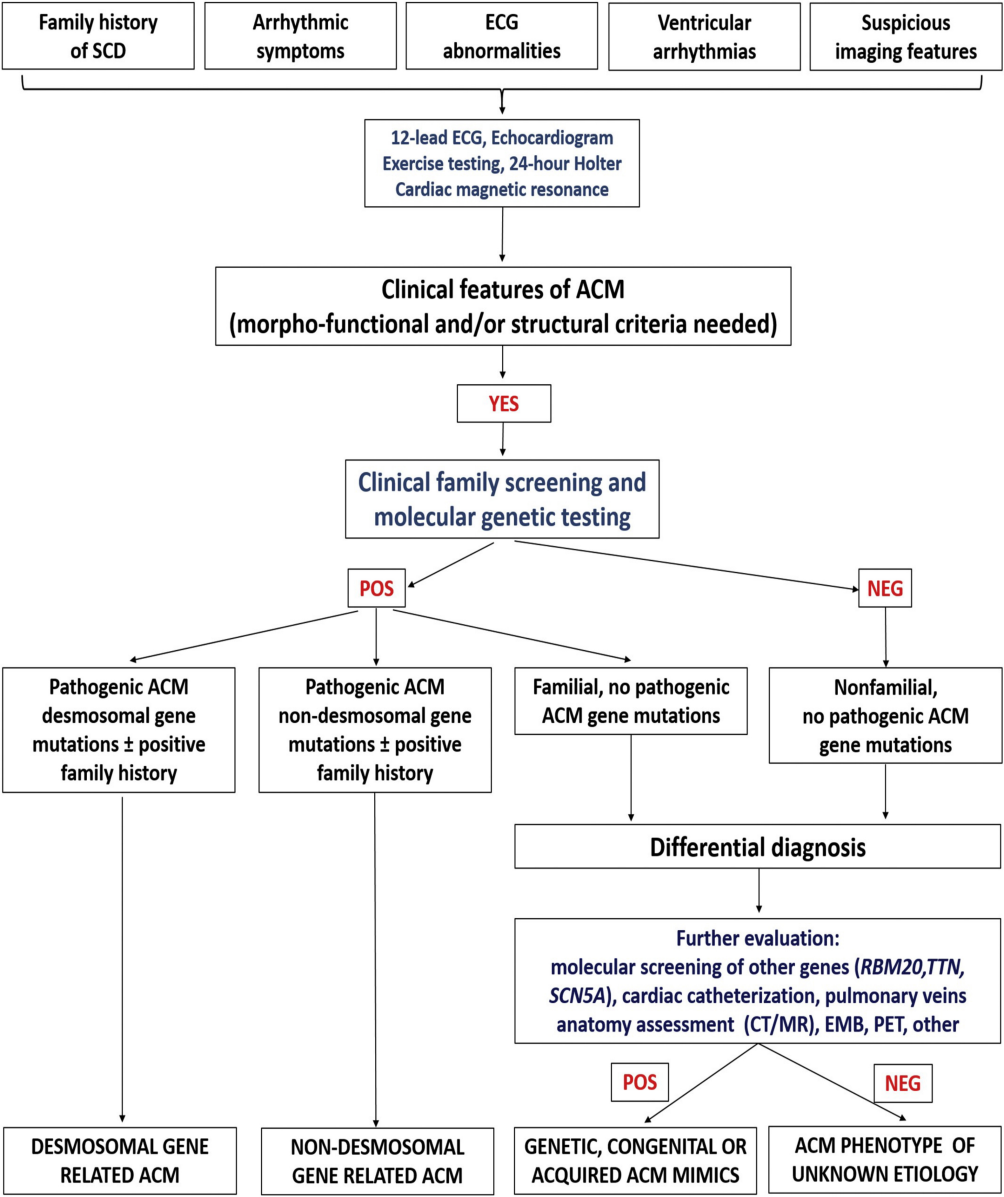
**Flow-chart for etiology assessment of ACM**. After the diagnosis is reached in a proband, cascade family screening and molecular genetic testing may allow to identify patients with identified gene mutation in a desmosomal or non-desmosomal gene. In patients with negative genetic testing, cascade family screening may allow to identify other affected family members: in this case, the diagnosis is a familial disease with still unknown genetic basis (so-called “gene elusive”). In case both genetic testing and family screening are negative, further testing may be performed to exclude phenocopies such as congenital heart disease or myocarditis. Adapted from Corrado *et al*. [19].

Once the diagnosis is reached, genetic testing and cascade family screening allow to classify the etiology of the disease into four categories: due to desmosomal gene mutation, due to non-desmomal gene mutation, familial but gene-elusive and non-genetic/non-familial. In this last case differential diagnosis with disease phenocopies must be considered, such as cardiac sarcoid (Fig. 2, Ref. [19]).

Therefore, the use of the 2020 International criteria can be considered as a three-step process. The first step is represented by a systematically research of signs of ACM through the multiparametric approach involving functional and structural ventricular abnormalities, tissue characterization findings, depolarization and repolarization ECG alterations, ventricular arrhythmias, family history and genetic findings. The second step is the identification of the specific phenotype and the likelihood of the disease according to the combination of the criteria fulfilled. The third step is to investigate the disease aetiology and differentiate ACM from phenocopies.

### 4.1 STEP 1: How Many Diagnostic Criteria are Satisfied?

I. Morpho-functional ventricular abnormalities

Such as in 2010 ITF criteria, echocardiography, CMR and angiography were indicated as possible tools for assessing morpho-functional ventricular abnormalities. However, in attempt to identify the disease earlier, any degree of RV dilation or dysfunction in association with regional wall motion abnormalities was considered a major RV criterion while the presence of regional wall motion abnormalities without RV dilatation or dysfunction was introduced as minor criterion. This finding is due to the regional nature of ACM that can affect the segmental contractility because of local fibro-fatty replacement, without compromising the global hemodynamics of the RV [14, 21]. At the same time, it has been classified as a minor criterion because of the misinterpretation of some normal variants of the RV wall motion [18].

The two morpho-functional criteria introduced for the LV are both minor because of the low specificity for diagnosing left-sided ACM variants. They include the detection of global LV systolic dysfunction with or without dilatation, and regional LV hypokinesia or akinesia. The use of strain echocardiography is recommended, because of the ability to detect subtle changes, especially in the early stages of the disease [22, 23].

Instead of using fixed cut-off values, the Padua criteria recommend the use of current reference values for cardiac chamber size and function, normalized for sex, age and body surface area, recommended by international societies of cardiovascular imaging [24, 25], and proper reference values for athletes, especially if engaged in sports associated with the greatest RV remodeling, such as canoeing and rowing [26].

II. Structural myocardial abnormalities

Fibrous or fibro-fatty replacement of myocardium is the pathological manifestation of ACM, and its detection through EMB has been indicated since 1994 [7]. However, because of its invasive nature with potential serious complications, the 2020 criteria have limited EMB indication to selected cases of non-familial ACM with negative genotyping to exclude phenocopies, such as myocarditis, sarcoidosis or DCM [15]. The demonstration of fibrous replacement of the RV myocardium in at least 1 sample, with or without fatty tissue, represents a major criterion. EMB is particularly helpful in ARVC, because of the peculiar topographic and histological features of the disease with fibro-fatty replacement reaching the subendocardium. A negative EMB do not exclude the diagnosis of ACM because of the possibility of sampling error. Moreover, the LV EMB is not indicated at present because the risk/benefit ratio is not yet known [27].

**Fig. 3.**
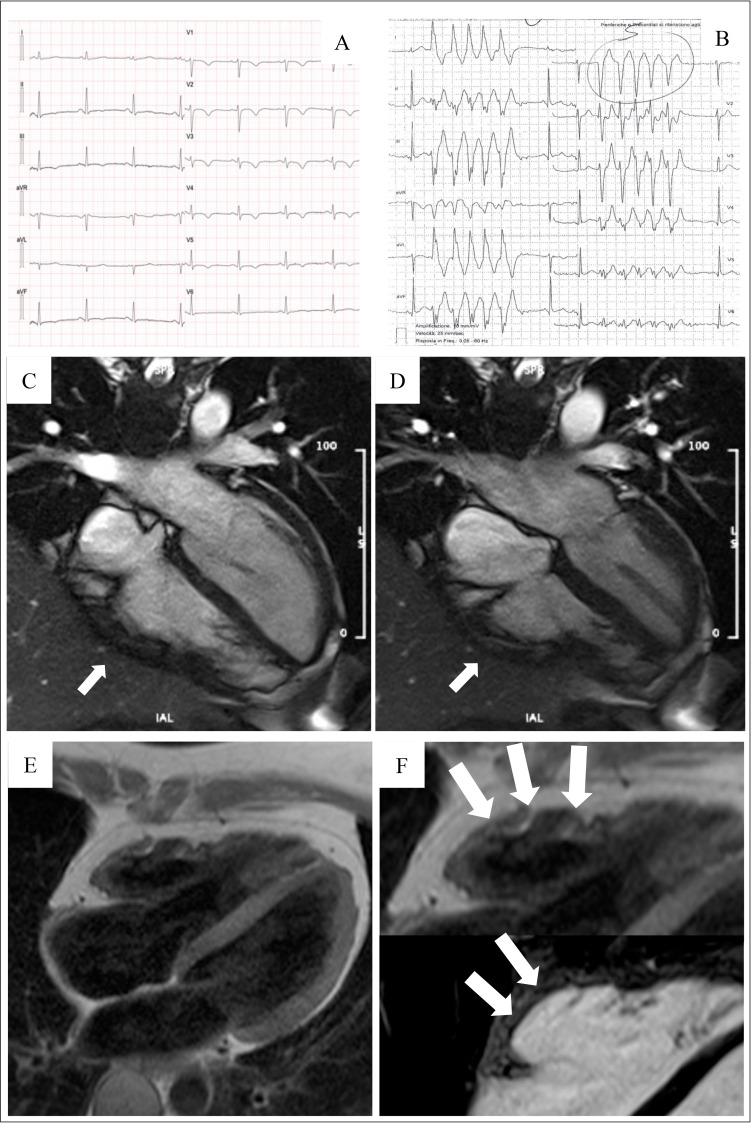
**Clinical features of ARVC**. Basal ECG, exercise testing ECG and CMR findings in a 38-year-old woman hospitalized for sustained VT. Basal ECG showed TWI in V1–V5 and flattened T wave in inferior leads (A). Exercise testing revealed frequent PVBs and a non-sustained VT with LBBB/superior axis morphology, originating from RV free wall (B). CMR revealed mild RV dilatation, moderate RV systolic disfunction, a wide peritricuspid aneurysm, with an extreme thinning of the wall (four-chamber cine view in diastolic phase, C, and systolic phase, D). The PD-TSE sequences revealed fatty infiltration of the RV wall, especially in the subtricuspid region (E, and magnified on the top of F). No RV LGE was identified, not even in the same regions of RV fatty infiltration, maybe because an extreme thinning of the RV wall (F on the bottom). The diagnosis was “definite ARVC”. ARVC, arrhythmogenic right ventricular cardiomyopathy; CMR, cardiac magnetic resonance; LBBB, left bundle branch block; LGE, late gadolinium enhancement; PD-TSE, positron density-turbo spin echo; PVBs, premature ventricular beats; RV, right ventricle; TWI, T wave inversion; VT, ventricular tachycardia. Adapted from Graziano *et al*. [31].

The introduction of non-invasive tissue characterization with CMR is one of the most important innovations of the 2020 criteria. The major CMR criterion is the presence of transmural LGE in at least 1 RV segment, confirmed in 2 orthogonal views. Currently, the diagnostic specificity of RV LGE is considered high, instead the sensitivity is low. This is due to the CMR technology characterized by a poor spectral resolution and suboptimal contrast/noise ratio in quantifying the thin RV wall [17, 28, 29, 30]. The highest specificity is reached when wall motion abnormalities and pre/post contrast signal alterations are considered together [29] (Fig. 3, Ref. [31]).

In the LV, the presence of a stria of LGE with a non-ischemic distribution (subepicardial and/or midmyocardial, most affecting the inferolateral region) in at least 1 LV Bull’s Eye segment, confirmed in 2 orthogonal views (excluding junctional LGE, that is considered non pathologic) is a major criterion. Moreover, the circumferential involvement of septum and LV free wall in short axis view is called “ring-like” pattern, and it is considered as highly specific for ALVC [32]. Nonetheless, at present there is no gold standard for differentiating non-ischemic LGE secondary to ACM or to other diseases such as myocarditis: for this reason, in the absence of concomitant RV involvement, the diagnosis of left-dominant ACM in a proband requires positive genetic testing (Fig. 1). Fatty tissue infiltration is often observed on dedicated CMR sequences, but it is not considered a diagnostic criterion in isolation because of its low specificity.

**Fig. 4.**
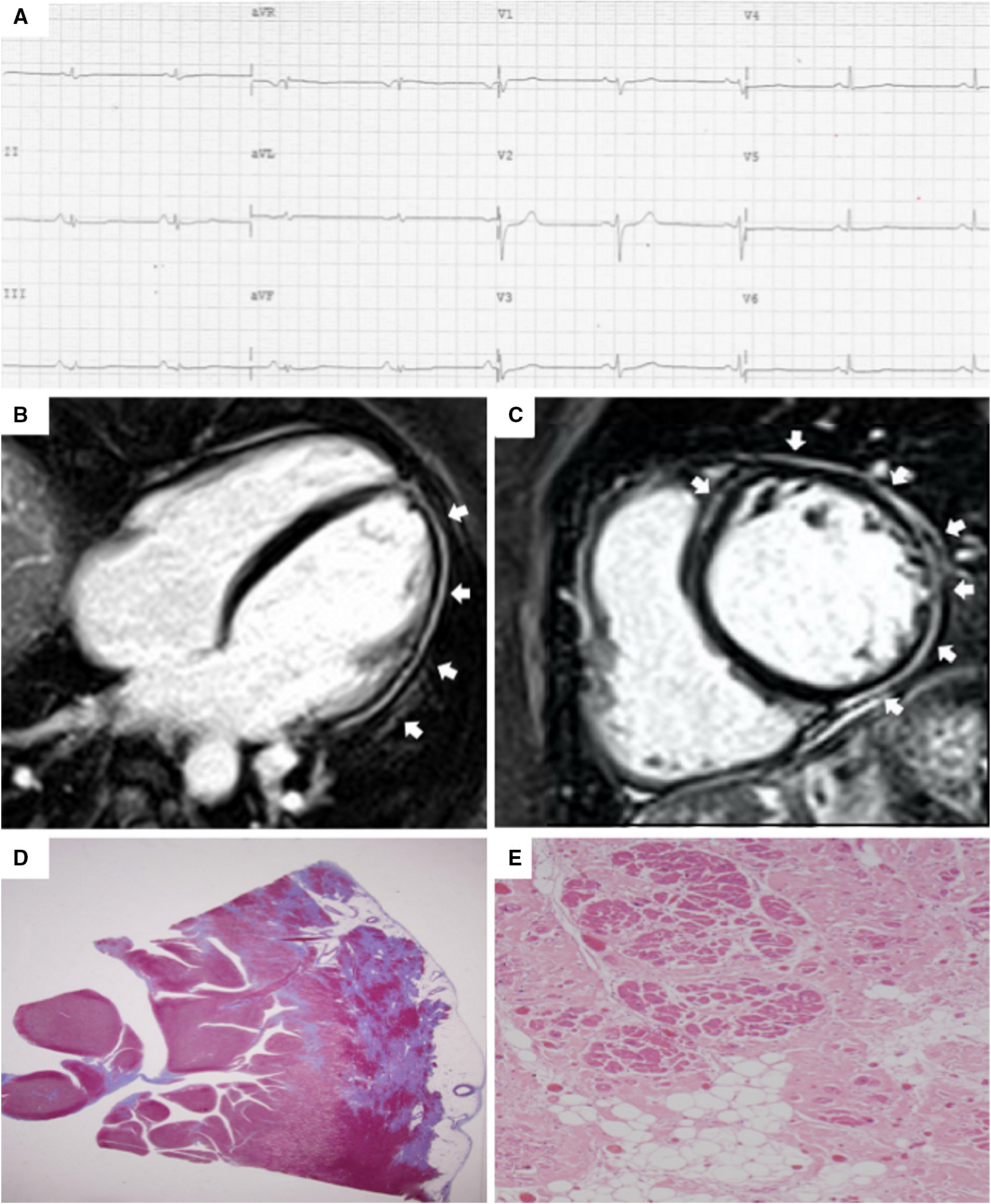
**Clinical and histopathological features of ALVC**. Basal ECG and CMR findings in a patient who underwent cardiac transplantation because of ALVC related to a DSP gene mutation. Basal ECG revealed low QRS voltages in limb leads and flattened T-waves in infero-lateral leads (A). Post-contrast sequences on CMR (four-chamber view, B, and short-axis view, C) revealed subepicardial LGE involving the anterior septum and the whole LV free wall (“ring like” pattern) from basal to apical regions. Histology in LV inferolateral region demonstrated fibrofatty myocardial replacement in the subepicardial layer (D); a magnification of residual myocytes embedded within fibrous and fatty tissue (hematoxylin and eosin stain) (E). The diagnosis was “definite ALVC”. ALVC, arrhythmogenic left ventricular cardiomyopathy; CMR, cardiac magnetic resonance; DSP, desmoplakin gene; LGE, late gadolinium enhancement; LV, left ventricle. Adapted from Cipriani *et al*. [14].

In the early stages of LV involvement, the typical non-ischemic distribution of fibro-fatty replacement sparing the subendocardial layer can explain the absence of wall motion abnormalities, dilatation, or dysfunction of the LV. Thereby, the absence of LV functional abnormalities on echo, cine-CMR or angiography cannot rule out LV involvement, and CE-CMR characterization plays a key role in detection of left-sided ACM [14, 16, 33, 34, 35, 36] (Fig. 4, Ref. [14]).

III. Repolarization abnormalities

As regards to repolarization abnormalities, the detection of T wave inversion (TWI) in right precordial leads (V1–V3) or beyond, or in V1–V2 only, remain major and minor criteria respectively. These findings require the absence of a complete right bundle branch block (RBBB). Otherwise, in the presence of RBBB, TWI in V1–V4 is a minor criterion. These criteria are valid in patients with complete pubertal development. A remarkable consideration is that TWI extension from right precordial leads (V1–V3) to left ones (V4–V6) is the expression of a more severe RV dilatation, with its displacement toward the axilla, rather than of LV involvement [34]. LV involvement can be only predicted with TWI in left precordial leads (V4–V6) in absence of complete LBBB, but it is a minor criterion because of its low specificity [15, 33, 34].

IV. Depolarization abnormalities

Signal averaged ECG is no more included among Padua criteria based on the experts’ opinion that they lacked specificity and showed low diagnostic accuracy [16]. Moreover, the epsilon wave in right precordial leads has been downgraded to minor criterion, because its identification and interpretation are highly influenced by ECG filtering and sampling rate, with a consequently large interobserver variability [37]. The ECG pattern in right precordial leads of a terminal activation duration (TAD) of the QRS ≥55 msec from the nadir of S wave to the end of QRS without a complete RBBB remains minor criterion, particularly if followed by TWI.

**Fig. 5.**
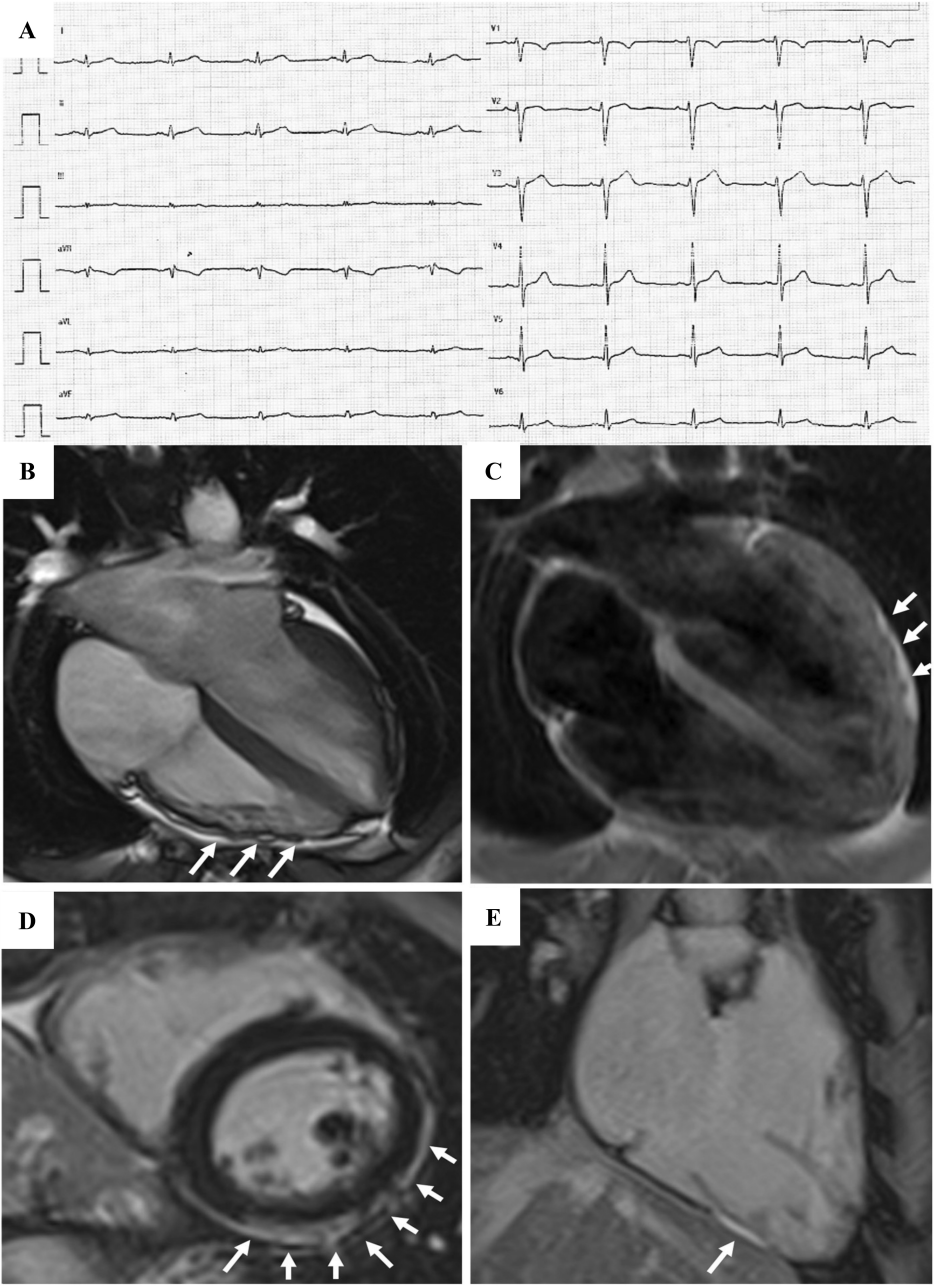
**Clinical features of biventricular ACM**. Basal ECG and CMR findings in a 28-year-old elite athlete. ECG revealed low QRS voltages in limb leads (A). Exercise testing demonstrated PVBs with a RBBB/superior axis morphology, isolated and in couples. CMR cine-sequences showed hypokinesis of the mid-apical lateral wall and multiple small bulging of the RV free wall (B, four-chamber view). The PD-TSE sequences revealed epicardial fatty infiltration of the lateral and inferior LV walls (C, four-chamber view). Post-contrast sequences on CMR demonstrated a subepicardial stria of LGE involving the antero-lateral, infero-lateral and inferior LV walls (D, short- axis view) and also the RV inferior wall (E, RV inflow-outflow view). The diagnosis was “definite biventricular ACM”. ACM, arrhythmogenic cardiomyopathy; CMR, cardiac magnetic resonance; LV, left ventricle; PVBs, premature ventricular beats; PD-TSE, proton density-turbo spin echo; RBBB, right bundle branch block; RV, right ventricle. Adapted from Zorzi *et al*. [38].

The presence of low QRS voltages in limb leads (peak-to-peak QRS amplitude <0.5 mV) is a predictor of LV involvement [14, 32, 35, 38]. The mechanism could be the reduction in generating of the electrical activity due to the fibro-fatty replacement of LV myocardial mass. It is a minor criterion in absence of other potential causes of low QRS voltages, such as emphysema, obesity, pericardial effusion, or inappropriate setting of low band-pass filters (<100 Hz) (Fig. 5, Ref. [38]).

V. Ventricular arrhythmias

ACM is characterized by premature ventricular beats (PVBs) with origin from or around the fibro-fatty tissue. PVBs are considered in terms of absolute number (>500 PVBs/24 h), complexity (sustained or non-sustained VT) and morphology on 12-ECG leads 24 h Holter monitoring or 12-ECG leads exercise test [39]. According to the 2020 Padua criteria, PVBs or VT with LBBB morphology originating from RV regions other than RVOT are more specific for ACM, so it is a major criterion. Instead, the LBBB/inferior axis morphology is less specific for ACM, because PVBs originating from RVOT are often idiopathic (minor criterion). The detection of PVBs with RBBB morphology suggests the origin from the LV, excluding the fascicular pattern (QRS <130 msec). RBBB/wide QRS/superior axis is the most common PVBs morphology in patient with LV scar involving the lateral or the infero-lateral wall, in biventricular ACM and ALVC [33, 34].

Electrophysiology study (EPS) is not considered a diagnostic criterion; however, it can be useful in selected patients for differential diagnosis. In particular, response to programmed ventricular stimulation and isoproterenol may allow to differentiate between idiopathic right-ventricular outflow tract (RVOT) VT or Brugada syndrome and the scar re-entrant VT of ACM. Addition of RV endocardial voltage mapping may be of incremental diagnostic value for differential diagnosis with idiopathic RVOT VT, as demonstration of low-voltage areas suggest the presence of fibro-fatty scars [16].

VI. Family history and molecular genetics

Compared to the 2010 ITF criteria, in the Padua criteria the category family history and molecular genetics has not changed. Moreover, the category is shared between RV and LV criteria. This is because the manifestation of ACM and the predominant ventricular involvement may vary among members of the same family and with the same gene mutation. However, more restricted indications for genotyping have been proposed to the new criteria, trying to avoid misinterpretation of molecular genetic results and misdiagnosis. The genetic test is recommended in probands with a definite biventricular or ARVC diagnosis, in order to screen family members [40]; it may be considered in borderline forms to reach the definite diagnosis, provided that the results of genetic test are interpreted by experts in ACM; it is mandatory to reach the diagnosis of non-familial ALVC to exclude phenocopies [16].

Major criteria are the detection of a pathogenic or likely pathogenic ACM gene mutation in the patient under evaluation, the history of a first-degree relative with ACM confirmed pathologically at autopsy or surgery or who reached the criteria necessary for ACM diagnosis. The minor criteria are the suspicion of ACM without confirmation in a first-degree relative, the suspicion of ACM in a first-degree relative who suddenly died before the age of 35, the confirmed diagnosis of ACM in a second-degree relative.

### 4.2 STEP 2: What is the Phenotype?

The second step is the identification of the specific ACM phenotype according to the number of criteria for the RV and LV involvement that are fulfilled. According to the 2020 ITF criteria, any diagnosis of ACM requires that at least 1 criterion from category I (morpho-functional abnormalities) or II (structural abnormalities) must be reached, either major or minor, and only these two categories are taken into consideration to classify the phenotypic variant.

If these criteria are only fulfilled for the RV, the diagnosis is the classical right-dominant variant (ARVC). Whereas, if the criteria are fulfilled for both RV and LV, the diagnosis is “biventricular” form. Moreover, it is possible to define the likelihood of disease according to the number of major and minor criteria reached from all categories. So, the diagnosis can be “definite” if either 2 major criteria, or 1 major and 2 minor criteria or 4 minor criteria are fulfilled, “borderline” if either 1 major and 1 minor criterion or 3 minor criteria are reached, and “possible” if either 1 major criterion or 2 minor criteria are satisfied.

The diagnosis of ALVC is reached in patients with structural LV abnormalities (major criterion) and no RV involvement, when a pathogenic or likely pathogenic ACM-causing gene mutation is identified. In this case, the diagnosis of ALVC is always definite. The need for positive genotyping testing is due to the possible overlap of these morpho-functional and structural findings with phenocopies, such as DCM, cardiac sarcoidosis or myocarditis.

### 4.3 STEP 3: What is the Etiology?

After the clinical diagnosis of ACM and the definition of the specific phenotype, the third step is to define the aetiology of ACM and to exclude phenocopies. This purpose can be reached thanks to molecular genetic testing and cascade family screening. Indeed, ACM is generally transmitted as an autosomal dominant trait, with variable expressivity and incomplete penetrance. So, the molecular genetic test can identify either desmosomal or non-desmosomal gene defects causing ACM.

Desmosomes are proteins forming the area composita of the intercalated disc that are structure crucial for electromechanical connection of cardiomyocytes and intracellular signaling cascades. These structures are also composed by adherens junctions, gap junctions and ion channels. Pathogenic mutations of gene encoding desmosomal proteins such as plakophilin (PKP2), desmoplakin (DSP), desmoglein (DSG2) and desmocollin (DSC2) are identified in ≈50% of patients with ARVC [40] and rarely (<1%) of gene encoding adherens junctional proteins such as N-cadherin (CDH2) and α-T-catenin (CTNNA3) [41, 42].

ACM left-sided variants are also associated with mutations in non-desmosomal genes encoding for ion channels and cytoskeletal components, such as lamin A/C (LMNA), filamin C (FLNC), transmembrane protein 43 (TMEM 43), desmin (DES), titin (TTN), phospholamban (PLN), the cardiac ryanodine receptor-2 (RYR2), sodium voltage-gated channel alpha subunit 5 (SCN5A) and transforming growth factor beta-3 (TGFβ-3) [40].

In case of a negative molecular genetic testing but positive clinical family screening, ACM is defined familial but “gene elusive”. Also in this case, the presence of affected relatives allows to rule out non-hereditary conditions mimicking ACM.

If both the genetic testing and the cascade clinical family screening for ACM are negative, it is essential to perform further evaluations in order to exclude mimics, both acquired (sarcoidosis, DCM, pulmonary artery hypertension, myocarditis, Chagas disease) and congenital (left-to right shunt or Ebstein anomaly) phenocopies.

## 5. Preliminary Clinical Experiences with the Use of Padua Criteria and Potential Limitations

In a cohort of 87 patients from the University of Padua who fulfilled the 2010 International Criteria for definite ACM, the application of the Padua criteria allowed to re-classify 51 of them as biventricular ACM because they also fulfilled either the morpho-functional or the structural criteria for LV involvement. Moreover, 5 of 15 patients with borderline diagnosis according to the 2010 ITF criteria were re-classified as definite ACM according to the Padua criteria. Finally, 9 patients with desmosomal-gene mutations but no signs of RV involvement met the major LV structural criterion and were thus re-classified as ALVC [14].

The additional value of the Padua criteria compared to the 2010 ITF was particularly evident among carriers of gene mutations characterized by predominant LV involvement such as desmoplakin, phospholamban and filamin-C genes. In a pooled analysis of patients with FLNC cardiomyopathy, 60 were diagnosed with ACM. Based on the 2010 ITF criteria, only a minority of patients fulfilled the criteria for definite ACM but according to the Padua criteria more than half of cases were diagnosed with definite left-dominant ACM [43]. Of 72 probands with DSP-gene mutations, Bariani *et al*. [44] showed that 26 had pure LV involvement and 7 biventricular involvement, but only 20 a classical ARVC. Overall, the number of patients reaching a definite diagnosis raised from 32 to 49 patients by using the 2020 Padua criteria compared with the 2010 ITF criteria. Moreover, Cicenia *et al*. [45] demonstrated that the application of the Padua criteria increased the sensitivity for ACM compared to the 2010 ITF criteria also in a small pediatric cohort, by demonstrating LV in half of the study sample.

These preliminary studies suggest the accuracy of the Padua criteria, but future studies on large populations are necessary to confirm their validity in diagnosing, and to assess their possible use for risk stratification and management of patients, especially in variants involving LV.

However, potential drawbacks of the Padua Criteria should be recognized. The first and most important is that they were proposed by a group of authors from the University of Padova and endorsed by several external experts, but they do not represent the result of an international consensus conference such as the 2010 ITF criteria. For this reason, they are still not universally accepted. There are then specific criteria that were based on experts’ opinion and thus require that their diagnostic accuracy is evaluated in the clinical practice. For example, evaluation of isolated wall motion abnormalities, particularly LV hypokinesia, is subject to high inter-observer variability; the acceptance of fibrotic changes in only one biopsy without any further quantification may potentially give rise to overestimation; and the exclusion of SAECG from Padua criteria was based on the experts’ opinion and was not supported by scientific data.

## 6. Conclusions

The development of the 2020 International criteria was a necessary step to improve the capability of diagnosing ACM. The most important innovation is the recognition and characterization of left-sided variants, which were underdiagnosed with the previous criteria. Because the typical ACM lesion is the subepicardial scar that may not cause wall motion abnormalities (particularly in the LV), the tissue characterization ability of CMR has become crucial. Preliminary data suggest that the diagnostic accuracy of ACM has improved thanks to the clinical use of the Padua criteria [14].

## References

[1] Corrado D, Link MS, Calkins H (2017). Arrhythmogenic Right Ventricular Cardiomyopathy. *New England Journal of Medicine*.

[2] Maron BJ (1988). Right Ventricular Cardiomyopathy: another Cause of Sudden Death in the Young. *New England Journal of Medicine*.

[3] Lancisi GM, Caput V, Musca (1736). *De motu cordis et aneurysmatibus. Opus posthumu, in duas partes divisum*.

[4] Marcus FI, Fontaine GH, Guiraudon G, Frank R, Laurenceau JL, Malergue C (1982). Right ventricular dysplasia: a report of 24 adult cases. *Circulation*.

[5] Thiene G, Nava A, Corrado D, Rossi L, Pennelli N (1988). Right Ventricular Cardiomyopathy and Sudden Death in Young People. *New England Journal of Medicine*.

[6] Richardson P, McKenna W, Bristow M, Maisch B, Mautner B, O’Connell J (1996). Report of the 1995 World Health Organization/International Society and Federation of Cardiology Task Force on the Definition and Classification of cardiomyopathies. *Circulation*.

[7] McKenna WJ, Thiene G, Nava A, Fontaliran F, Blomstrom-Lundqvist C, Fontaine G (1994). Diagnosis of arrhythmogenic right ventricular dysplasia/cardiomyopathy. Task Force of the Working Group Myocardial and Pericardial Disease of the European Society of Cardiology and of the Scientific Council on Cardiomyopathies of the International Society and Federation of Cardiology. *British Heart Journal*.

[8] Hamid MS, Norman M, Quraishi A, Firoozi S, Thaman R, Gimeno JR (2002). Prospective evaluation of relatives for familial arrhythmogenic right ventricular cardiomyopathy/dysplasia reveals a need to broaden diagnostic criteria. *Journal of the American College of Cardiology*.

[9] Marcus FI, McKenna WJ, Sherrill D, Basso C, Bauce B, Bluemke DA (2010). Diagnosis of arrhythmogenic right ventricular cardiomyopathy/dysplasia: Proposed Modification of the Task Force Criteria. *European Heart Journal*.

[10] Corrado D, Thiene G (2006). Arrhythmogenic Right Ventricular Cardiomyopathy/Dysplasia: clinical impact of molecular genetic studies. *Circulation*.

[11] Sen-Chowdhry S, Syrris P, McKenna WJ (2005). Genetics of Right Ventricular Cardiomyopathy. *Journal of Cardiovascular Electrophysiology*.

[12] Awad MM, Calkins H, Judge DP (2008). Mechanisms of Disease: molecular genetics of arrhythmogenic right ventricular dysplasia/cardiomyopathy. *Nature Clinical Practice Cardiovascular Medicine*.

[13] Norman M, Simpson M, Mogensen J, Shaw A, Hughes S, Syrris P (2005). Novel Mutation in Desmoplakin Causes Arrhythmogenic Left Ventricular Cardiomyopathy. *Circulation*.

[14] Cipriani A, Bauce B, De Lazzari M, Rigato I, Bariani R, Meneghin S (2020). Arrhythmogenic Right Ventricular Cardiomyopathy: Characterization of Left Ventricular Phenotype and Differential Diagnosis with Dilated Cardiomyopathy. *Journal of the American Heart Association*.

[15] Corrado D, Basso C, Judge DP (2017). Arrhythmogenic Cardiomyopathy. *Circulation Research*.

[16] Corrado D, van Tintelen PJ, McKenna WJ, Hauer RNW, Anastastakis A, Asimaki A (2020). Arrhythmogenic right ventricular cardiomyopathy: evaluation of the current diagnostic criteria and differential diagnosis. *European Heart Journal*.

[17] Perazzolo Marra M, Rizzo S, Bauce B, De Lazzari M, Pilichou K, Corrado D (2015). Arrhythmogenic right ventricular cardiomyopathy. Contribution of cardiac magnetic resonance imaging to the diagnosis. *Herz*.

[18] Rastegar N, Burt JR, Corona-Villalobos CP, te Riele AS, James CA, Murray B (2014). Cardiac MR Findings and Potential Diagnostic Pitfalls in Patients Evaluated for Arrhythmogenic Right Ventricular Cardiomyopathy. *RadioGraphics*.

[19] Corrado D, Perazzolo Marra M, Zorzi A, Beffagna G, Cipriani A, Lazzari M (2020). Diagnosis of arrhythmogenic cardiomyopathy: The Padua criteria. *International Journal of Cardiology*.

[20] Corrado D, Zorzi A, Cipriani A, Bauce B, Bariani R, Beffagna G (2021). Evolving Diagnostic Criteria for Arrhythmogenic Cardiomyopathy. *Journal of the American Heart Association*.

[21] Marra MP, Leoni L, Bauce B, Corbetti F, Zorzi A, Migliore F (2012). Imaging study of ventricular scar in arrhythmogenic right ventricular cardiomyopathy: comparison of 3D standard electroanatomical voltage mapping and contrast-enhanced cardiac magnetic resonance. *Circulation: Arrhythmia and electrophysiology*.

[22] Haugaa KH, Basso C, Badano LP, Bucciarelli-Ducci C, Cardim N, Gaemperli O (2017). Comprehensive multi-modality imaging approach in arrhythmogenic cardiomyopathy—an expert consensus document of the European Association of Cardiovascular Imaging. *European Heart Journal - Cardiovascular Imaging*.

[23] Borgquist R, Haugaa KH, Gilljam T, Bundgaard H, Hansen J, Eschen O (2014). The diagnostic performance of imaging methods in ARVC using the 2010 Task Force criteria. *European Heart Journal - Cardiovascular Imaging*.

[24] Lang RM, Badano LP, Mor-Avi V, Afilalo J, Armstrong A, Ernande L (2015). Recommendations for cardiac chamber quantification by echocardiography in adults: an update from the American Society of Echocardiography and the European Association of Cardiovascular Imaging. *European Heart Journal: Cardiovascular Imaging*.

[25] Petersen SE, Khanji MY, Plein S, Lancellotti P, Bucciarelli-Ducci C (2019). European Association of Cardiovascular Imaging expert consensus paper: a comprehensive review of cardiovascular magnetic resonance normal values of cardiac chamber size and aortic root in adults and recommendations for grading severity. *European Heart Journal - Cardiovascular Imaging*.

[26] D’Ascenzi F, Anselmi F, Piu P, Fiorentini C, Carbone SF, Volterrani L (2019). Cardiac Magnetic Resonance Normal Reference Values of Biventricular Size and Function in Male Athlete’s Heart. *JACC: Cardiovascular Imaging*.

[27] Basso C, Ronco F, Marcus F, Abudureheman A, Rizzo S, Frigo AC (2008). Quantitative assessment of endomyocardial biopsy in arrhythmogenic right ventricular cardiomyopathy/dysplasia: an in vitro validation of diagnostic criteria. *European Heart Journal*.

[28] Perazzolo Marra M, Cipriani A, Rizzo S, De Lazzari M, De Gaspari M, Akrami N (2021). Myocardial Tissue Characterization in Arrhythmogenic Cardiomyopathy: Comparison Between Endomyocardial Biopsy and Cardiac Magnetic Resonance. *JACC: Cardiovascular Imaging*.

[29] Aquaro GD, Barison A, Todiere G, Grigoratos C, Ait Ali L, Di Bella G (2016). Usefulness of Combined Functional Assessment by Cardiac Magnetic Resonance and Tissue Characterization Versus Task Force Criteria for Diagnosis of Arrhythmogenic Right Ventricular Cardiomyopathy. *The American Journal of Cardiology*.

[30] Tandri H, Saranathan M, Rodriguez ER, Martinez C, Bomma C, Nasir K (2005). Noninvasive detection of myocardial fibrosis in arrhythmogenic right ventricular cardiomyopathy using delayed-enhancement magnetic resonance imaging. *Journal of the American College of Cardiology*.

[31] Graziano F, Zorzi A, Cipriani A, De Lazzari M, Bauce B, Rigato I (2022). The 2020 “Padua Criteria” for Diagnosis and Phenotype Characterization of Arrhythmogenic Cardiomyopathy in Clinical Practice. *Journal of Clinical Medicine*.

[32] Augusto JB, Eiros R, Nakou E, Moura-Ferreira S, Treibel TA, Captur G (2020). Dilated cardiomyopathy and arrhythmogenic left ventricular cardiomyopathy: a comprehensive genotype-imaging phenotype study. *European Heart Journal: Cardiovascular Imaging*.

[33] Sen-Chowdhry S, Syrris P, Prasad SK, Hughes SE, Merrifield R, Ward D (2008). Left-Dominant Arrhythmogenic Cardiomyopathy: an under-recognized clinical entity. *Journal of the American College of Cardiology*.

[34] De Lazzari M, Zorzi A, Cipriani A, Susana A, Mastella G, Rizzo A (2018). Relationship between Electrocardiographic Findings and Cardiac Magnetic Resonance Phenotypes in Arrhythmogenic Cardiomyopathy. *Journal of the American Heart Association*.

[35] Zorzi A, Perazzolo Marra M, Rigato I, De Lazzari M, Susana A, Niero A (2016). Nonischemic Left Ventricular Scar as a Substrate of Life-Threatening Ventricular Arrhythmias and Sudden Cardiac Death in Competitive Athletes. *Circulation: Arrhythmia and Electrophysiology*.

[36] Zorzi A, Rigato I, Pilichou K, Perazzolo Marra M, Migliore F, Mazzotti E (2016). Phenotypic expression is a prerequisite for malignant arrhythmic events and sudden cardiac death in arrhythmogenic right ventricular cardiomyopathy. *Europace*.

[37] Platonov PG, Calkins H, Hauer RN, Corrado D, Svendsen JH, Wichter T (2016). High interobserver variability in the assessment of epsilon waves: Implications for diagnosis of arrhythmogenic right ventricular cardiomyopathy/dysplasia. *Heart Rhythm*.

[38] Zorzi A, Bettella N, Tatangelo M, Del Monte A, Vessella T, Poscolieri B (2022). Prevalence and clinical significance of isolated low QRS voltages in young athletes. *Europace*.

[39] Corrado D, Drezner JA, D’Ascenzi F, Zorzi A (2020). How to evaluate premature ventricular beats in the athlete: critical review and proposal of a diagnostic algorithm. *British Journal of Sports Medicine*.

[40] Hoorntje ET, te Rijdt WP, James CA, Pilichou K, Basso C, Judge DP (2017). Arrhythmogenic cardiomyopathy: pathology, genetics, and concepts in pathogenesis. *Cardiovascular Research*.

[41] Mayosi BM, Fish M, Shaboodien G, Mastantuono E, Kraus S, Wieland T (2017). Identification of Cadherin 2 (CDH2) Mutations in Arrhythmogenic Right Ventricular Cardiomyopathy. *Circulation: Cardiovascular Genetics*.

[42] van Hengel J, Calore M, Bauce B, Dazzo E, Mazzotti E, De Bortoli M (2013). Mutations in the area composita protein αT-catenin are associated with arrhythmogenic right ventricular cardiomyopathy. *European Heart Journal*.

[43] Celeghin R, Cipriani A, Bariani R, Bueno Marinas M, Cason M, Bevilacqua M (2022). Filamin-C variant-associated cardiomyopathy: a pooled analysis of individual patient data to evaluate the clinical profile and risk of sudden cardiac death. *Heart Rhythm*.

[44] Bariani R, Cason M, Rigato I, Cipriani A, Celeghin R, De Gaspari M (2022). Clinical profile and long-term follow-up of a cohort of patients with desmoplakin cardiomyopathy. *Heart Rhythm*.

[45] Cicenia M, Cantarutti N, Adorisio R, Silvetti MS, Secinaro A, Ciancarella P (2022). Arrhythmogenic cardiomyopathy in children according to “Padua criteria”: Single pediatric center experience». *International Journal of Cardiology*.

